# Inactivation of HMGCL promotes proliferation and metastasis of nasopharyngeal carcinoma by suppressing oxidative stress

**DOI:** 10.1038/s41598-017-11025-2

**Published:** 2017-09-20

**Authors:** Wenqi Luo, Liting Qin, Bo Li, Zhipeng Liao, Jiezhen Liang, Xiling Xiao, Xue Xiao, Yingxi Mo, Guangwu Huang, Zhe Zhang, Xiaoying Zhou, Ping Li

**Affiliations:** 1 Department of Pathology, First Affiliated Hospital of Guangxi Medical University, Nanning, China; 2 Department of Radiotherapy, First Affiliated Hospital of Guangxi Medical University, Nanning, China; 3 Department of Otolaryngology-Head & Neck Surgery, First Affiliated Hospital of Guangxi Medical University, Nanning, China; 4 Department of Research, Affiliated Tumor Hospital of Guangxi Medical University, Nanning, China; 5 Life Science Institute, Guangxi Medical University, Nanning, China

## Abstract

Altered metabolism is considered as a hallmark of cancer. Here we investigated expression of 3-hydroxy-3-methylglutaryl-coenzyme A (HMG-CoA) 2 lyase (HMGCL), an essential enzyme in ketogenesis, which produces ketone bodies by the breakdown of fatty acids to supply energy, in nasopharyngeal carcinoma (NPC). The expression of HMGCL was silenced in NPC tissue. Downregulation of HMGCL in NPC was associated with low intracellular β-hydroxybutyrate (β-HB) production, thereby reducing reactive oxygen species (ROS) generation. Ectopic expression of HMGCL restored β-HB level, associated with suppressed proliferation and colony formation of NPC cells *in vitro* and decreased tumorigenicity *in vivo*. HMGCL suppressed the migration and invasion of NPC cells *in vitro* via mesenchymal-epithelial transition. Furthermore, extracellular β-HB supply suppressed the proliferation and migration of NPC cells. Both intra- and extracellular β-HB exerting a suppressive role in NPC depends on ROS generation. Ketogenesis may be impaired in NPC cells due to lack of HMGCL expression, suggesting that it may be a promising target in NPC therapy.

## Introduction

Nasopharyngeal carcinoma (NPC) is a malignancy frequently originating in the slit-like nasopharyngeal recess called fossa of Rosenmüller. NPC is associated with distinct geographical, racial and ethnic distribution^1^. The incidence of NPC is less than 1/100,000 people worldwide, but the cancer is endemic in Southeast Asia and southern China, with an incidence of 20 to 30/100,000 people^2^. Multiple factors are involved in the carcinogenesis of NPC, including genetic susceptibility, environmental factors and Epstein-Barr virus (EBV) infection^1,2^. NPC is conventionally treated with radiotherapy and early-stage NPC can be cured this way, but a significant number of patients still show local recurrence and distant metastases, so novel strategies for NPC therapy are needed^3^.

The development and progression of malignancy is accompanied by altered metabolism of tumor cells, considered as a hallmark of cancer^4^. This metabolic reprogramming of tumor cells facilitates their adaption to the tumor microenvironment, which provides the needed energy to sustain their malignant behavior, including accelerated proliferation, apoptosis resistance, evasion of immune attack, and maintenance of a cancer stem cell state^5–7^. One of the well-known strategies used by tumor cells is the Warburg effect, whereby tumor cells “ferment” glucose to lactate via aerobic glycolysis to generate ATP despite abundant oxygen^8^. The metabolic switch of NPC could be regulated by EBV-encoded products^9,10^, cellular genes^11^, long non-coding RNAs (LnRNAs)^12^, and microRNAs^13^, targeting the glucose transporters and glycolytic enzymes.

The intermediate product of glycolysis, acetyl-CoA, is a main ingredient for fatty acid (FA) synthesis. Intracellular long-chain FAs are metabolized to be stored as neutral lipids. FAs are esterified to produce triglycerides and phospholipids or used for energy production and transfer, then FAs are oxidated. The oxidative metabolism of FAs yields CO_2_ as its end product or FAs are incompletely metabolized to ketone bodies, which can be secreted and used by neighbor cells^14,15^. Fast-proliferating tumor cells have a high requirement for lipids (FAs and cholesterol), which are used mainly for biosynthesis of structural components of the cellular membrane, as well as for production of energy during nutrient deprivation^16^.

Various kinds of tumors increase their capacity to synthesize FAs and store them as lipid droplets (LDs)^17–19^. The accumulation of LDs is associated with the expression of other “stemness” markers in colorectal and glioma cancer stem cells^18,20^. We observed that LDs accumulate in human NPC cell lines and primary tumor tissues^21,22^. EBV infection can modulate FA synthesis by upregulating the expression of FA synthase and lipogenesis^23^. Preclinical and clinical studies have demonstrated an anticancer effect of drugs interfering with FA β-oxidation or lipid synthesis^24^. However, the molecular mechanisms involved in altered lipid metabolism in NPC remain unclear.

Another metabolic conversion of FA is ketogenesis. Ketone bodies, that is, β-hydroxybutyrate (β-HB), acetoacetate (AcAc), and acetone, are mainly produced in the liver. Besides occurring in hepatocytes, ketone body production and release has been shown in normal epithelial cells and tumors^25–27^. The ketogenesis and function of ketone bodies in tumorigenesis and tumor progression has been under debate. Certain kinds of tumor cells use ketone bodies in proliferation and metastasis^28^ (e.g., increasing the intracellular level of acetoacetate specifically promotes activation of MEK-ERK signaling in melanoma^27^). However, in some malignancies such as brain and gastric cancers, tumor cells cannot effectively use ketone bodies for energy. Extracellular ketone bodies have strong anti-proliferative and pro-apoptotic effects in several cancers, including pancreatic and gastric cancer, as well as EBV-positive lymphoblasts^29–31^. In addition, ketone bodies affect intracellular oxidative stress^32^, which is crucial for the survival and function of tumor cells^33^.

3-Hydroxy-3-methylglutaryl-coenzyme A (HMG-CoA) 2 lyase (HMGCL) catalyzes the cleavage of HMG-CoA into acetyl-CoA and acetoacetate to mediate the rate-limiting step in the metabolic processing of ketone bodies for energy production^34^. To date, only a few studies have verified the role of HMGCL in human cancers. HMGCL was found upregulated in androgen-independent prostate cancer cells and BRAF-mutated melanoma^26,27^. In breast cancer, HMGCL and other enzymes associated with ketone-body production were preferentially expressed in the tumor stroma^35^. The oncogenic Kaposi’s sarcoma-associated herpesvirus was shown to interfere with transcription of HMGCL directly by targeting its promoter region^36^.

Here, we investigated the expression and possible role of HMGCL in NPC. Because HMGCL is a key enzyme in ketogenesis, we analyzed the effect of both intracellular and extracellular ketone-body production on the malignant behavior of NPC cells and examined the possible mechanisms involved.

## Results

### Identification of differentially expressed ketogenesis genes in NPC tumors

**Figure 1:**
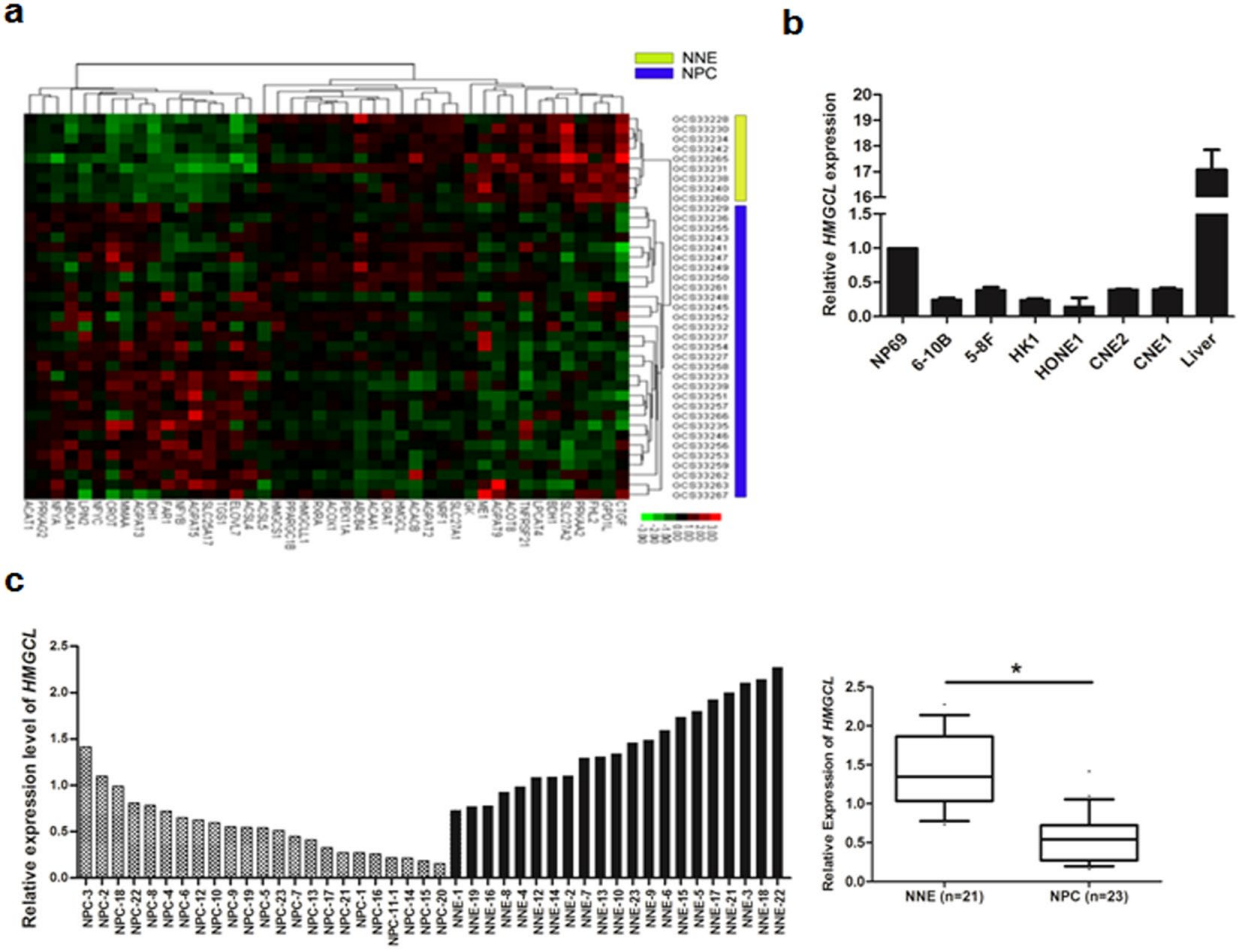
Transcription of *HMGCL* is downregulated in nasopharyngeal carcinoma (NPC). (**a**) A heat map showing 41 differentially expressed genes involved in lipid metabolism in NPC (n = 30) compared with normal nasopharyngeal epithelium (NNE, n = 9) based on cDNA microarray data (GSE 12452). (**b**) Real-time RT-PCR analysis of the mRNA level of *HMGCL* in 6 NPC cell lines and a non-cancerous nasopharyngeal epithelial cell line NP69. Human liver tissue was used as a positive control. (**c**) Real-time RT-PCR analysis of the mRNA level of *HMGCL* in NPC primary tumors (n = 23) and NNE tissue (n = 21). Boxes indicate 25 to 75 percentile, horizontal line indicates the mean, and bars indicate 10 and 90 percentiles. (*p < 0.05).

To understand the reprogramming of genes participating in regulating lipid metabolism in NPC, we used microarray data from the GEO database (GSE 12452), including 30 cases of NPC tissue and 9 cases of normal nasopharyngeal epithelium (NNE). We found 41 genes with differential expression between the 2 tissue types. Two genes, *HMGCL* and *BDH1*, both involved in the ketogenesis pathway, were significantly downregulated in NPC tissue (Fig. 1a), which suggests altered ketone-body metabolism. We further analyzed microarray data from the GSE 53819 database including 18 cases of NPC and 18 cases of NNE controls. In this screen, we found 2 genes involved in ketogenesis, HMGCL and ACAT1 (data not shown). The HMGCL gene was selected for further study.

### The expression of HMGCL is downregulated in NPC

To confirm our microarray data, we firstly investigated the transcriptional level of *HMGCL* in NPC samples by quantitative real-time PCR. As compared with an immortalized normal nasopharyngeal epithelial cell line (NP69), 6 NPC cell lines (CNE1, CNE2, HONE1, HK1, 5-8 F and 6-10 B) showed reduced mRNA level of HMGCL (Fig. 1b). As well, the expression of the *HMGCL* gene was downregulated in 23 NPC primary tumors but easily detected in all 21 NNE samples (Fig. 1c). In summary, in all 23 NPC cases tested, the expression of *HMGCL* was significantly lower than the mean HMGCL expression in NNE samples (p < 0.05, Fig. 1c).

**Figure 2:**
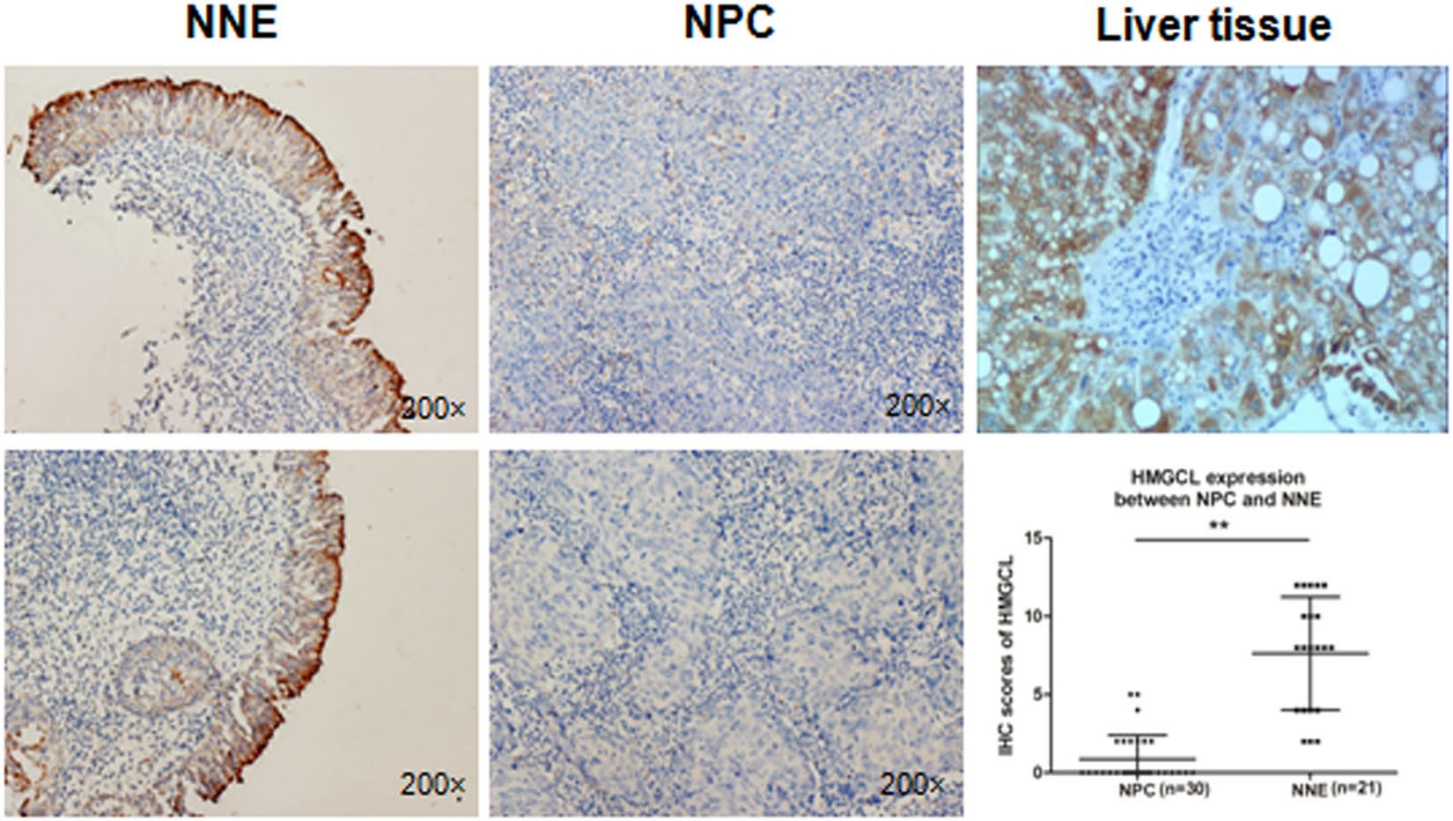
The expression of HMGCL is downregulated in NPC. Immunohistochemical staining of HMGCL in NPC (n = 30) and NNE tissue (n = 21). Representative images are immunostaining results for 2 NNE and 2 NPC samples (magnification ×200). The scored HMGCL expression is shown in the scatter plot. Human liver tissue was used as a positive control. (**p < 0.01).

In addition, we measured the protein levels of HMGCL in NPC tissue by immunohistochemical (IHC) staining. HMCGL was localized in the cytoplasm of cells and was highly expressed in the NNE layer (n = 21) (Fig. 2) but was almost absent in NPC tissue (n = 30). This finding further supports our finding that *HMGCL* is transcriptionally inactivated in NPC.

### HMGCL increases intracellular level of β-hydroxybutyrate (β-HB) and acetoacetate (AcAc) and generates ROS in NPC cells

**Figure 3:**
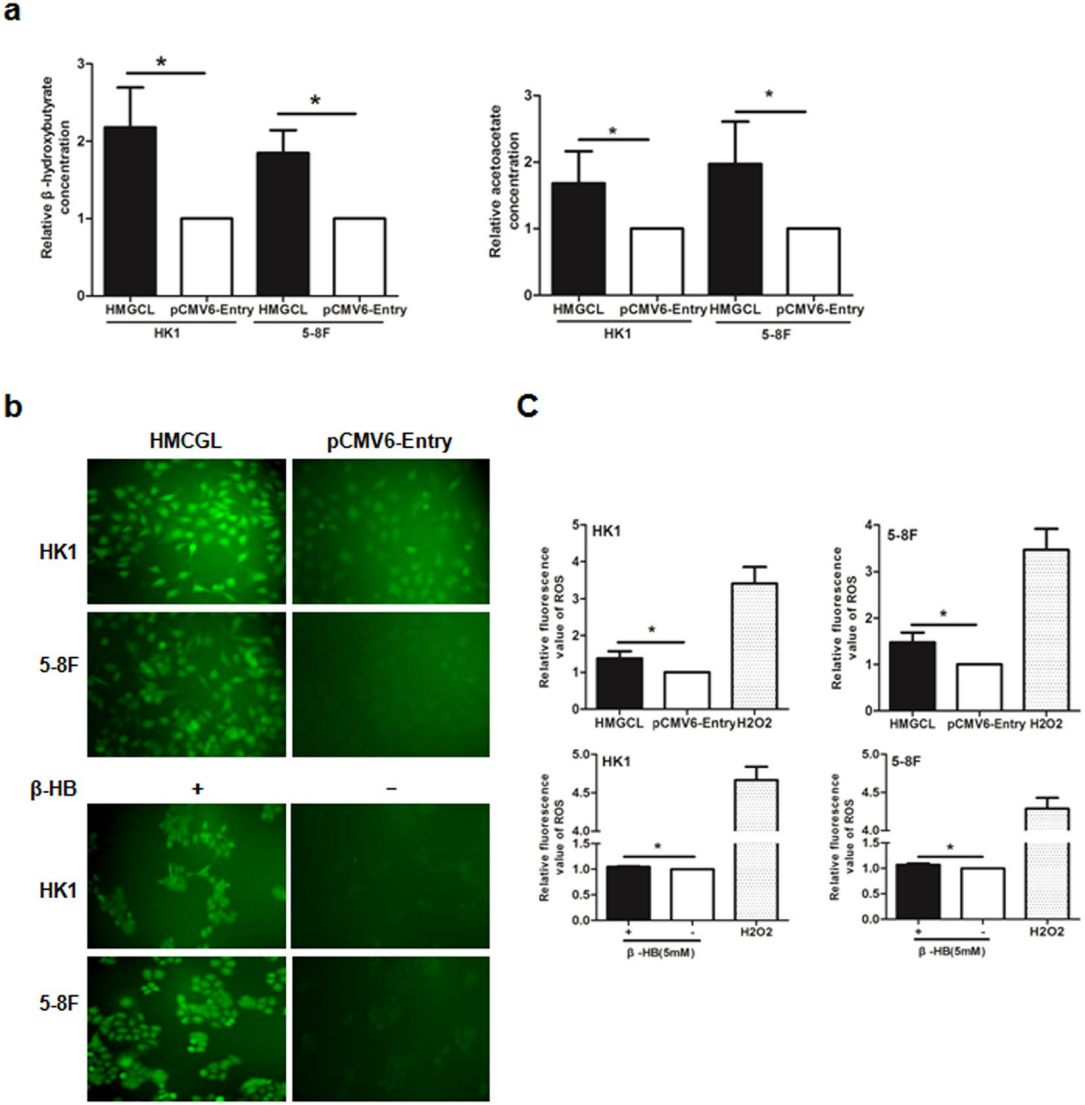
HMGCL elevates intracellular level of β-hydroxybutyrate (β-HB) and acetoacetate (AcAc) to increase reactive oxygen species (ROS) generation in NPC cells. (**a**) Relative concentration of intracellular β-HB and AcAc in HMGCL-HK1 and pCMV6-Entry-HK1 cells. Data are mean ± SD (n = 3). (**b**) Detection of ROS by DCFH-DA probe with green fluorescent signals in HMGCL-HK1/5-8 F and pCMV6-Entry-HK1/5-8 F cells. Parental HK1 and 5-8 F cells were treated with 5 mM β-HB for 24 h before ROS detection (magnification × 200). (**c**)The fluorescent signals were also detected by a plate reader. Cells treated with H_2_O_2_ (50 μg/ml) for 30 min was used as a positive control of ROS detection. Data are mean ± SD (n = 3). (*p < 0.05).

To understand the possible function of HMGCL, we stably transfected NPC cells with an HMGCL construct or the corresponding empty-vector pCMV6-Entry construct. We assessed the relative concentration of intracellular β-HB and AcAc, the main components of ketone bodies in HMGCL-HK1 and pCMV6-Entry-HK1 cells. Both intracellular β-HB and AcAc level was significantly higher with HMGCL-HK1 than pCMV6-Entry-HK1 (Fig. 3a), which suggests that HMGCL expression directly contributes to ketogenesis in NPC cells.

Because ketone bodies could increase ROS production^37^, we further analyzed the association between intracellular/extracellular β-HB and ROS level. β-HB generated by ectopic expression of HMGCL as well as extracellular β-HB treatment elevated ROS production in NPC cells (Fig. 3b,c), so the effect of HMGCL expression in NPC cells may be mediated by ROS.

### Exogenous expression of HMGCL suppresses NPC cell proliferation *in vitro* and *in vivo*

**Figure 4:**
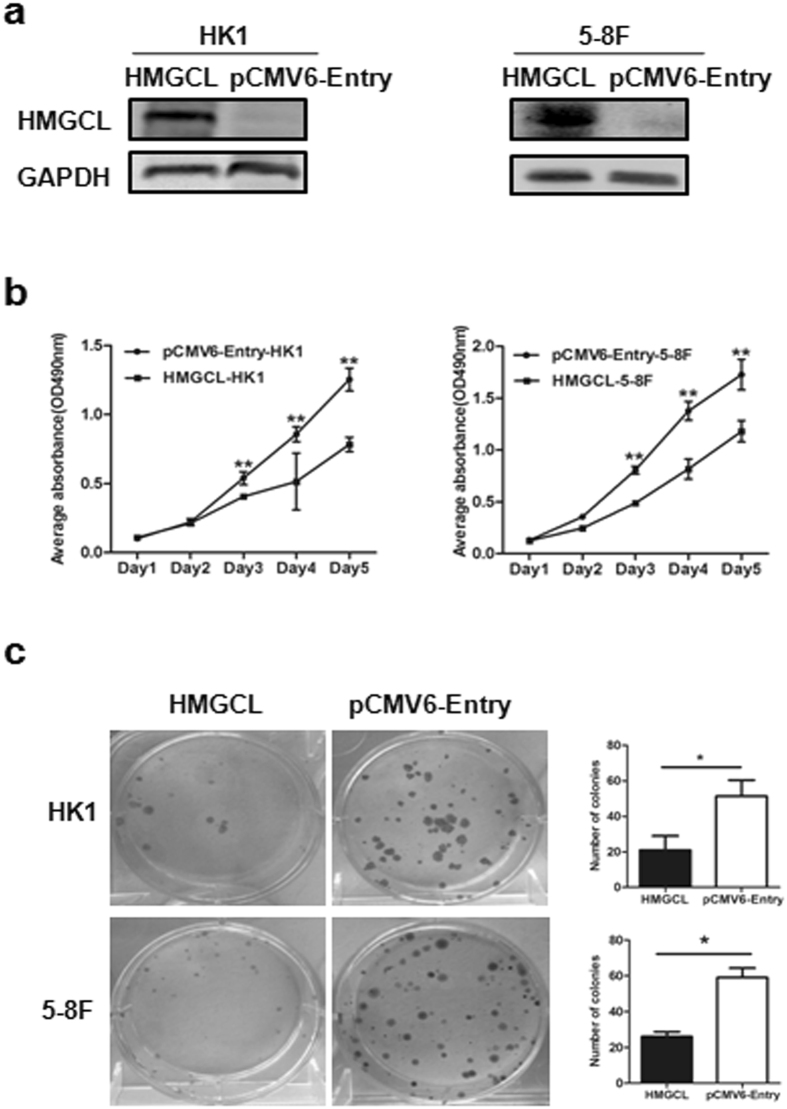
Exogenous expression of *HMGCL* suppresses proliferation and colony formation of NPC cells *in vitro*. (**a**) The expression of *HMGCL* in HK1 and 5-8 F cell lines was confirmed by Western blot. (**b**) MTT assay of growth curves for HK1 and 5-8 F cells (OD = 490 nm). (**c**) Colony formation assay of HK1 and 5-8 F cells for 12 days. Data are mean ± SD (n = 3) (*p < 0.05; **p < 0.01).

To explore the functional role of *HMGCL* in cell growth, we compared the proliferation of *HMGCL*-HK1 and *HMGCL*-5-8F cells to corresponding control cell lines pCMV6-Entry-HK1 and pCMV6-Entry-5-8F by MTT assay. HMGCL was expressed in *HMGCL*-HK1 and *HMGCL*-5-8F cells (Fig. 4a). HK1/5-8 F cells transfected with HMGCL grew significantly slower than pCMV6-Entry-HK1/5-8 F control cells (Fig. 4b, P < 0.05). Fewer colonies were observed in *HMGCL*-HK1/5-8 F than control cells (Fig. 4c).

**Figure 5:**
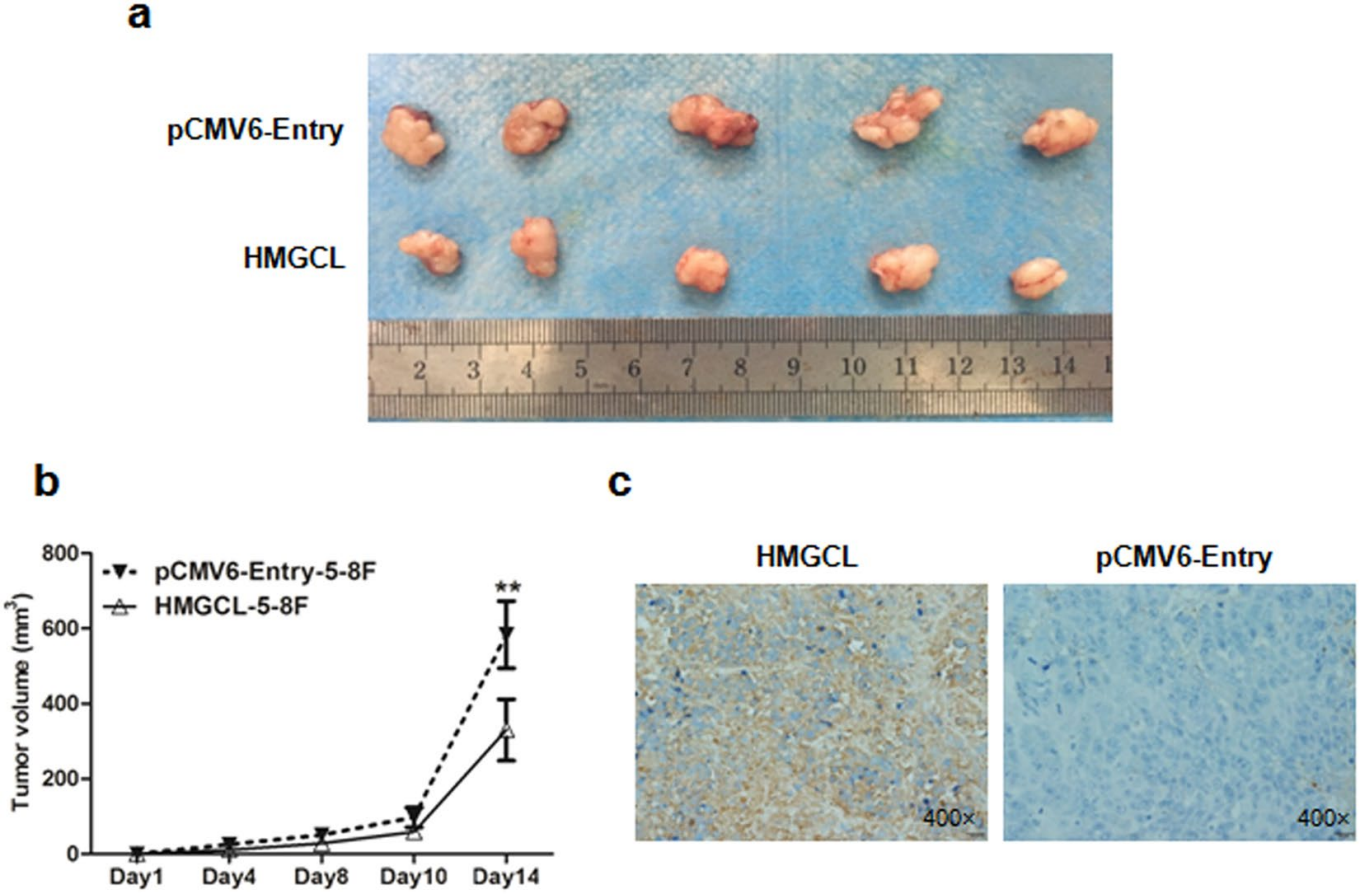
HMGCL suppresses tumorigenicity of NPC cells *in vivo*. (**a**) Xenografts from HMGCL-5-8F and pCMV6-Entry-5-8F cell injection were removed at day 14 after inoculation in nude mice. (**b**) Volume of the tumors was measured every 2 days after inoculation. (**c**) Immunohistochemical staining of HMGCL in removed tumors (magnification ×200). (*p < 0.05).

To determine whether the effect of HMGCL expression on tumor suppression could be reproduced *in vivo*, we subcutaneously injected HMGCL-5-8F and pCMV6-Entry-5-8F cells in opposite flanks of nude mice. Tumor volume was smaller with inoculation of HMGCL-5-8F than pCMV6-Entry-5-8F cells (Fig. 5a,b). IHC staining of the tumors confirmed the increased expression of HMGCL in tumors from HMGCL-5-8F injection (Fig. 5c). Taken together, exogenous expression of HMGCL may inhibit the growth of NPC cells both *in vitro* and *in vivo*.

### Exogenous expression of HMGCL inhibits NPC cell migration and invasion by reversing the epithelial–mesenchymal transition (EMT)

**Figure 6:**
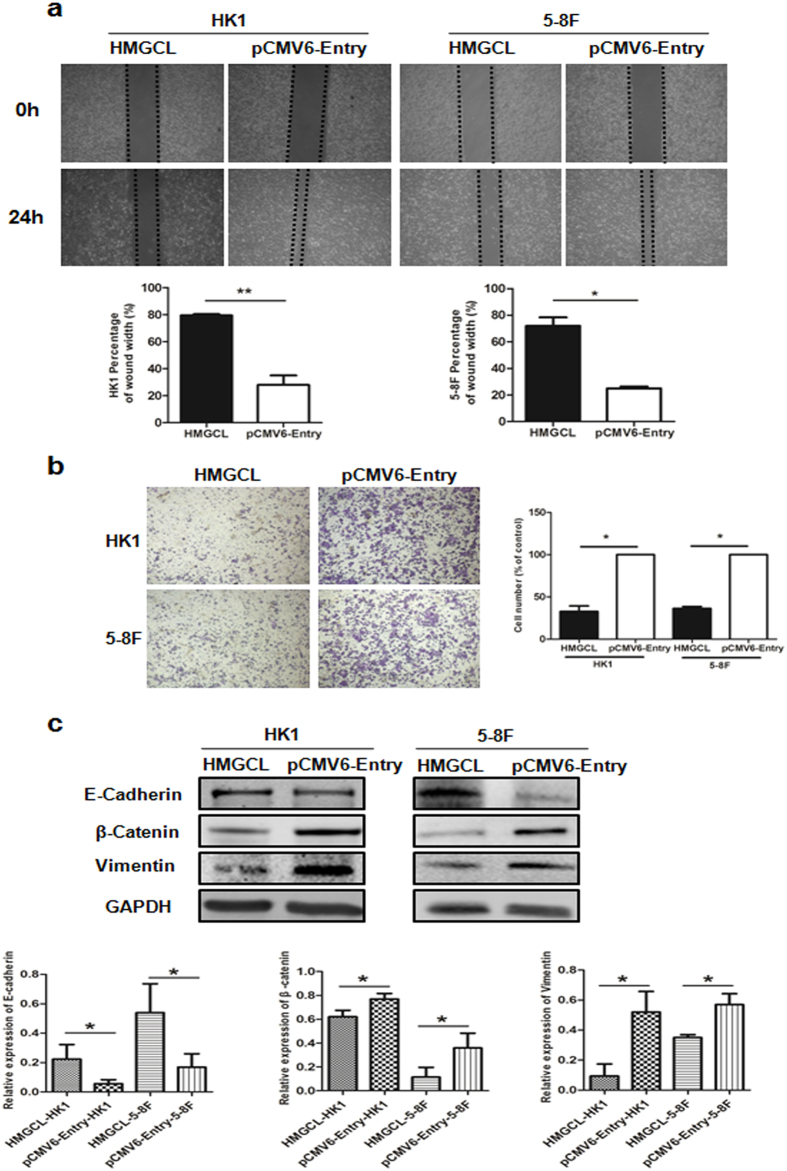
HMGCL inhibits NPC cell migration and invasion by reversing the epithelial–mesenchymal transition (EMT). (**a**) Wound healing assays of HMGCL-HK1/5-8 F and pCMV6-Entry-HK1/5-8 F cell lines. The gap was photographed and measured at 0 and 24 h. The percentage of wound width for each sample was calculated as [width (μm) at 24 h]/[width (μm) at 0 h] × 100%. (**b**) Transwell assay of invasive capacity of HMGCL-HK1/5-8 F and pCMV6-Entry-HK1/5-8 F cells. The blue dots represent the invading cells stained with crystal violet. The number of invading cells were counted and shown in the bar graph. (**c**) Western blot analysis of the expression of three key molecules involved in EMT, E-cadherin, β-catenin and Vimentin. GAPDH was an internal control. Data are mean ± SD (n = 3). (*p < 0.05; **p < 0.01).

A metabolic shift, including glycolysis and ROS generation, contributes to NPC metastasis^38,39^. To investigate the role of HMGCL in the metastatic potential of NPC cells, we used 2D and 3D model systems to determine the capacity for migration and invasion, respectively. In scratch assay, the gap closure was slower for HMGCL-HK1/5-8 F than pCMV6-Entry-HK1/5-8 F cells, indicating that HMGCL expression retarded the migration of NPC cells (Fig. 6a). Transwell assay also confirmed that overexpression of HMGCL markedly inhibited the invasive capacity of HK1 and 5-8 F cells (Fig. 6b).

To reveal the underlying mechanism of HMGCL, we evaluated its effect on key EMT-associated proteins. The expression of E-cadherin was upregulated in HMGCL-HK1/5-8 F cells and that of β-catenin and vimentin was decreased (Fig. 6c). Accordingly, decreasing HMGCL expression in NPC cell lines may be involved in the EMT process and promote migration and invasion. Overexpression of HMGCL may reverse the EMT process, thereby inhibiting the metastasis potential of NPC cells.

### ROS inhibitor promotes NPC cell growth and migration *in vitro*

**Figure 7:**
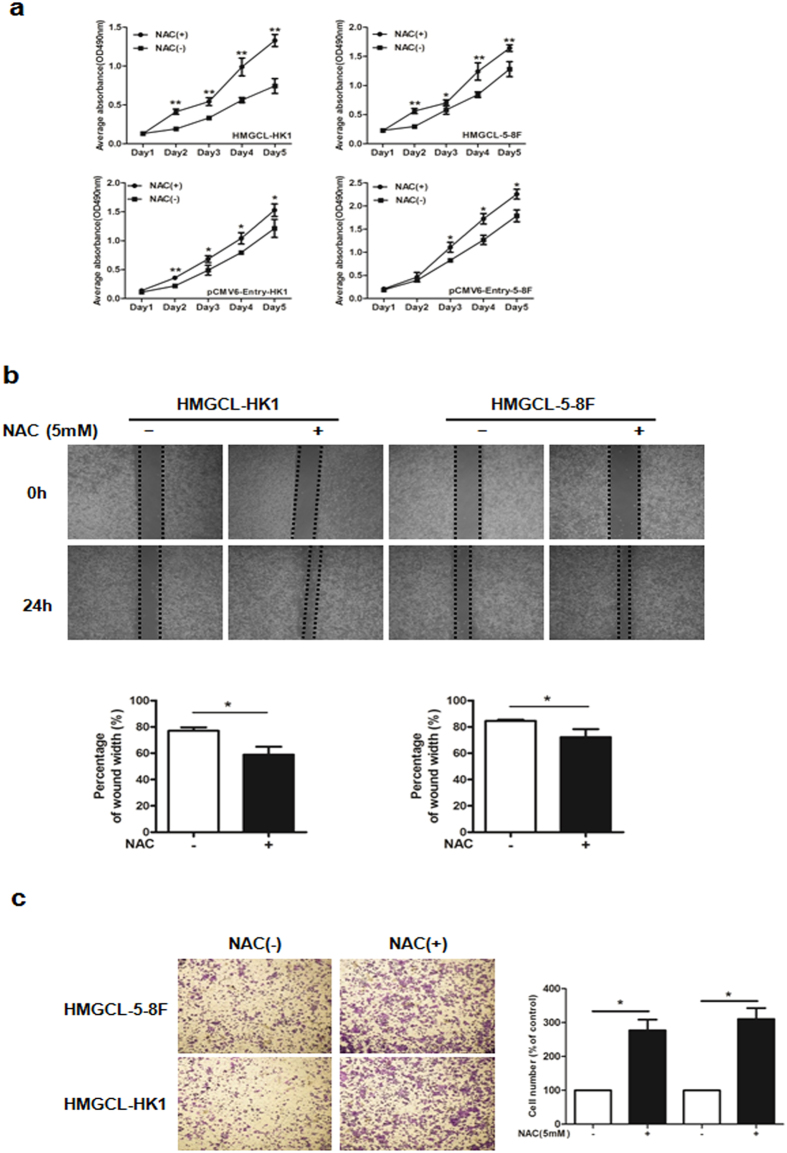
ROS inhibitor N-acetyl cysteine (NAC) accelerates NPC cell growth and migration *in vitro*. (**a**) The effect of NAC (5 mM) on proliferation of HMCGL-HK1/5-8 F and pCMV6-HK1/5-8 F cell lines measured by MTT assay. The effect of NAC (5 mM) on migratory (**b**) and invasive capacity (**c**) of HMCGL-HK1/5-8 F cells assessed by wound healing assay and transwell assay, respectively. Data are mean ± SD (n = 3) (*p < 0.05; **p < 0.01).

To verify whether the inhibitory effect of HMGCL on NPC cells depends on ROS generation, we used the ROS inhibitor N-acetyl cysteine (NAC) to reduce the level of ROS in HMGCL-HK1/5-8 F cells. NAC treatment increased the proliferation of HMGCL-HK1/5-8 F cells (Fig. 7a) and reducing the ROS level in HMGCL-HK1/5-8 F cells accelerated their migratory capacity (Fig. 7b). The invasion of HMGLC-5-8F was enhanced by NAC (Fig. 7c). The inhibitory effect of HMGCL on the growth and metastasis of NPC cells was reversed by treatment with the ROS inhibitor by inducing EMT (Fig. S1a), which suggests that ROS is a key mediator of HMGCL activity.

### Extracellular β-HB inhibits NPC cell proliferation and migration depends on increasing ROS levels

**Figure 8:**
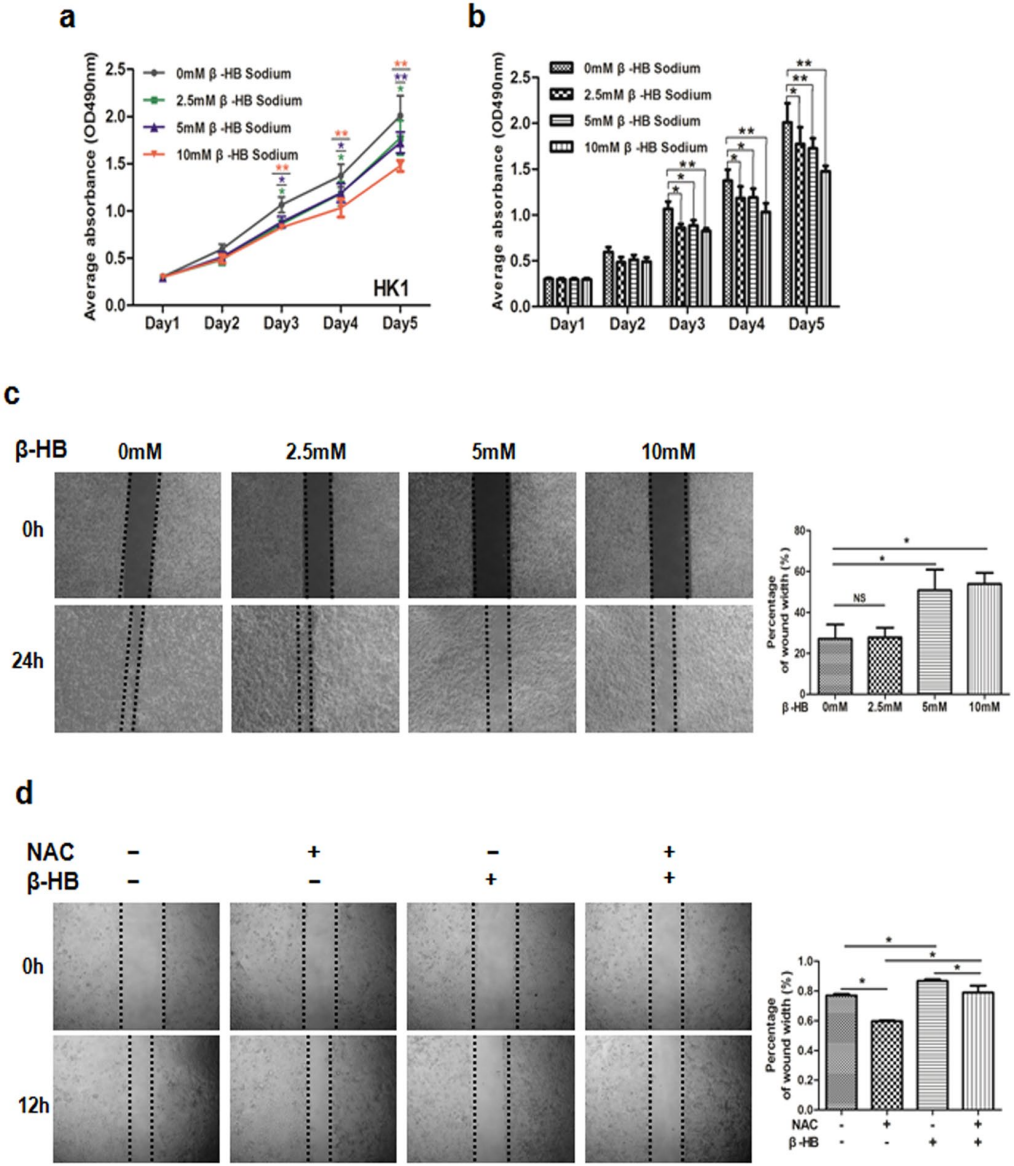
β-HB treatment inhibits NPC cell proliferation and migration. (**a**,**b**) MTT assay was performed to measure the proliferation of HK1 cells after β-HB treatment at 0 mM, 2.5 mM, 5 mM and 10 mM. (**c**) Motility of HK1 cells with various concentrations of β-HB. (**d**) Motility in HK1 cells with 5 mM NAC and 5 mM β-HB, alone or combined. Data are mean±SD (n = 3) (*p < 0.05; **p < 0.01).

Both intracellular and extracellular β-HB could stimulate ROS generation in NPC cells (Fig. 3b). Hence, we further evaluated the effect of extracellular β-HB on growth and mobility of NPC cells. Growth and motility of HK1 cells were dose-dependently suppressed with β-HB treatment (Fig. 8a–c). Consistent with previous results, that β-HB treatment attenuates the migration of 5-8 F cells by reversing the EMT (Fig. S1b). However, AcAc did not play significant role as β-HB (Fig. S2). Furthermore, the combined treatment of β-HB and NAC increased the migration of HK1 cells (Fig. 8d). Therefore, the inhibitory effect of extracellular β-HB on NPC cells was similar to that of intracellular β-HB, relying on ROS generation.

## Discussion

Many human cancers share metabolic alterations. In general, tumor cells metabolize glucose, lactate, pyruvate, hydroxybutyrate, acetate, glutamine, and FAs at much higher rates than do their non-tumor equivalents, which maximizes meeting the fundamental needs of proliferating cells^24^. Targeting metabolic alteration has become a new avenue of therapeutic intervention in tumors.

By using an “in silico” approach, we analyzed the differential global transcription of genes involved in metabolic pathways in NPC compared to NNE tissue. HMGCL, a key enzyme in ketogenesis, was downregulated in NPC tissue. Both HMGCL transcription and protein expression was decreased in NPC cell lines and primary tumors. Besides of NPC, we also analyzed the RNA-sequencing database from the cancer genome atlas (TCGA) and found *HMGCL* transcription downregulated in several kinds of cancer, including head and neck, kidney and colon cancers (data not shown). Another ketogenic enzyme, HMGCS2, was found downregulated in colon cancer and associated with de-differentiation of colonic epithelium^40^. These further suggest that altered ketogenesis may be a common feature in tumor tissue.

Intracellular long chain FAs undergo metabolism via two major pathways: esterification to build up FA esters (triglycerides, phospholipids) or oxidation to CO_2_ and ketone bodies. In lung cancer, the mutation of *KRAS* promotes β-oxidation of FAs and the conversion of FAs into the acyl-CoA of FAs, which serve as substrates for lipid synthesis^41^. We previously found greater accumulation of LDs in NPC cells than NNE cells, which suggests elevated esterification of FAs in NPC^21^. In addition to downregulating HMGCL, this metabolic pathway might contribute to the reduced ketogenesis in NPC. Conversely, lack of induction of the ketogenesis enzyme HMGCS2 might contribute to impaired FA β-oxidation^42^.

In investigating the functional consequences of HMGCL downregulation in NPC, we detected an increased level of intracellular β-HB, a downstream product of HMGCL, in NPC cells stably transfected with HMGCL. Ketone-body metabolism may be critical for tumor progression and metastasis in NPC.

Ketogenic diet, as a novel approach in the treatment of cancer, has been discussed for a long time, but the clinical data are still limited^43^. It has been shown that anti-tumor immunity is suppressed by inflammatory factor IL-6 by decreasing ketogenesis^44^. In the contrary, a therapeutic ketogenic diet can enhance tumor-reactive immune responses^45^. Ketogenic diet is suggested to have anti-cancer effect *in vitro* and *in vivo* ^31,46^, and could be applied for management of brain tumors in both preclinical and clinical settings^47^. Hence, ketogenesis may be an undesirable metabolic characteristic of NPC cells and a target for NPC therapy.

We also found that β-HB treatment of NPC cells significantly decreased their viability, motility and proliferation and that this decrease could be reversed by the ROS antioxidant NAC. Thus, ROS is a major downstream mediator of the effects of intracellular ketone bodies, and the inhibition of proliferation caused by ketone bodies depends on ROS in NPC. In NPC tumors, every cell carries the EBV genome in a latent state. A low level of ROS might be essential for maintaining EBV infection. More than 120 genes are encoded by EBV, but only a few are expressed in NPC latent infection. This situation reduces the immunogenicity of the latently EBV-infected cells, thereby allowing them to evade immune attack. A recent study reported that increased ROS production could trigger the reactivation of EBV from latency in NPC cells^48^. We also showed that knocking down a reductase, GLRX3, impaired the proliferation and metastasis of NPC cells by elevating the level of ROS^39^. Hence, inhibition of ketongenesis by downregulating HMGCL may be one of the mechanisms involved in modulating EBV latent infection in NPC cells, thus contributing to tumorigenesis and progression by reducing the immunogenicity of NPC cells. This association of EBV latency and HMGCL expression in NPC needs further investigation.

Epigenetic mechanisms such as DNA methylation, histone modifications and microRNAs, which are altered in the EBV genome and in host cells, may underlie the initiation and progression of NPC^49^. Dysregulated expression of histone deacetylaces (HDACs) results in aberrant gene expression, thereby altering cellular functions, including malignant transformation^50^. HDACs are also involved in maintaining the EBV latent cycle. HDAC inhibitors (HDACi) are potent inducers of EBV reactivation, which is critical for expression of lytic proteins, thereby providing novel targets for therapy as well as mediating the enhanced killing of cancer cells when used alone or with additional anti-cancer agents in EBV-associated malignancies^51,52^. β-HB belongs to the endogenous inhibitors of HDACs. In addition, a lower level of β-HB impairs the differentiation of intestinal cells^53^, which suggests an important role of ketone bodies in the development of epithelium. Therefore, elevating the level of β-HB by targeting a ketogenic gene might be a promising approach for NPC.

In summary, HMGCL was downregulated in NPC cells and tumor tissues. Overexpression of HMGCL in NPC cells increased the levels of ketone bodies and ROS, thereby inhibiting cell proliferation, suppressing EMT and reducing NPC cell invasion and migration. Our findings reveal a novel mechanism of NPC tumorigenesis that metabolism of ketone bodies may affect NPC cells by disrupting the redox balance of the intracellular environment. Thus, manipulating ketone-body metabolism might be a new area for drug discovery and dietary interventions for prevention and treatment of NPC.

## Materials and Methods

### Ethics statement

Ethical permission for this study was granted by the Research Ethics Committee of the First Affiliated Hospital of Guangxi Medical University, China (documents no.2016-KY-050). Written informed consent was obtained from all patients and healthy participants. All methods were performed in accordance with the relevant guidelines and regulations.

### Study samples

NPC cell lines CNE1, CNE2, HONE1, HK1, 5-8 F and 6-10B were maintained in DMEM medium (Invitrogen, Carlsbad, CA, USA) supplemented with 10% fetal bovine serum (FBS; Invitrogen) in an atmosphere with 5% CO_2_ at 37 °C^54–57^. The nonmalignant nasopharyngeal epithelial cell line NP69 was grown in defined keratinocyte serum-free medium (Invitrogen)^58^.

In total, 53 NPC primary tumor biopsies were collected from newly diagnosed and untreated NPC patients at the Department of Otolaryngology-Head and Neck Surgery, First Affiliated Hospital of Guangxi Medical University (Nanning, China). A total of 42 normal nasopharyngeal epithelium (NNE) tissue samples with chronic inflammation were included as controls. Overall, 23 NPC and 21 NNE samples were used for RNA extraction, and 30 NPC biopsy and 21 NNE samples were formalin-fixed and paraffin-embedded.

### Antibodies, plasmids and reagents

Primary antibodies were for HMGCL (HPA004727; Sigma-Aldrich, St. Louis, MO, USA); β-catenin (sc-376841; Santa Cruz Biotechnology, Santa Cruz, CA, USA); and E-cadherin (#3195 P), Vimentin (#5741 P) and GAPDH (#5174 P; all Cell Signaling, Beverly, MA, USA). The secondary antibody 680RD goat anti-mouse and IRDye^®^800CW goat anti-rabbit antibody were from LI-COR Biosciences (Lincoln, NE, USA).

The full-length coding sequence for *HMGCL* subcloned into the pCMV6-Entry vector was from Origene (Rockville, MD, USA). Two NPC cell lines (HK1 and 5-8 F) were transfected with the pCMV6-Entry-*HMGCL* plasmid and empty-vector pCMV6-Entry, respectively, by using X-tremeGENE transfection reagents (#06366236001, Roche, Mannheim, Germany). Stable clones were obtained by selection in 400 µg/ml G418 (#G8160, Solarbio, Beijing, China) for 2 weeks and maintained in medium with 250 µg/ml G418. The ROS inhibitor N-acetyl-L-cysteine (NAC, #A7250) was from Sigma-Aldrich.

### Quantitative Real-time PCR (qRT-PCR)

Total RNA was extracted from cells and tissues by using TRIzol reagent (Invitrogen, Carlsbad, Carlsbad, CA, USA) and cDNA synthesis involved use of the Prime Script RT reagent kit (Invitrogen, Carlsbad, CA, USA). The expression of *HMGCL* mRNA was determined by using Fast start universal green master mix in the ABI 7500 Real Time PCR system (Applied Biosystems, USA). Glyceraldehyde-3-phosphate dehydrogenase (GAPDH) was an internal control. The primer sequences were for *HMGCL*-F: 5′-ACCACCAGCTTTGTGTCTCC-3′, *HMGCL*-R: 5′-GAGGCAGCTCCAAAGATGAC-3′; and *GAPDH*-F: 5′-GCACCGTCAAGGCTGAGAAC-3′, *GAPDH*-R: 5′-TGGTGAAGACGCCAGTGGA-3′.

### Immunohistochemical staining

Tissues were cut into 3-μm-thick sections and incubated for 1 h with 3% hydrogen peroxide to eliminate endogenous peroxidase activity after deparaffinization and rehydration. After antigen retrieval, sections were incubated with HMGCL antibody at 4 °C overnight, then secondary antibody for 1 h at room temperature. Subsequently, 3,3′-diaminobenzidine (DAB) reagent (ZLI-9018, ZSGB-BIO, Beijing) was used for peroxidase reaction and hematoxylin was used for counterstaining. Images were acquired under a microscope (Olympus C-5050, Japan). The immunohistochemistry results were independently evaluated by two pathologists who were blinded to sample status. The intensity of HMGCL staining was scored and graded as described^59^.

### β-hydroxybutyrate (β-HB) detection assay

Stably transfected cell lines pCMV6-Entry-HK1 and *HMCGL*-HK1 were grown in 100-mm dishes in serum-free DMEM medium for 48 h. Then cells were lysed with RIPA buffer (Beyotime, Jiangsu, China). Intracellular β-HB level was determined by using a β-HB colorimetric assay kit (#700190, Cayman Chemical, Ann Arbor, MI, USA). The concentration of β-HB was normalized to that of protein in the lysate.

### ROS detection

Intracellular ROS was detected by use of a kit (S0033, Beyotime, Jiangsu, China). Briefly, adherent cells were incubated with DCFH-DA probe in serum-free DMEM medium at 37 °C for 30 min, then washed with PBS three times and visualized under a fluorescent microscope or detected by a fluorescent reader (Biotek synergy HTX, USA).

### Cell proliferation assay

Cells (2 × 10^3^ cells per well) were seeded into 96-well plates and allowed to grow for 5 days to determine a growth curve. Cell density was examined every 24 h by use of 3-(4,5-dimethylthiazol-2-yl)-2,5-diphenyl tetrazolium bromide (MTT) assay (M1025, Solarbio, Beijing), in a plate reader with absorbance at OD490 nm (iMark, Bio-Rad, Hercules, CA, USA). Each experiment was performed in quintuplicate.

### Colony formation assay

Cells (100 per well) were seeded in 6-well plates. The medium was changed every 3 days. After 14 days, Giemsa-stained colonies were photographed and counted by use of Quantity One v4.4.0 (Bio-Rad, USA). The experiment was performed in triplicate.

### *In vivo* tumorigenicity assay

Five 4-week-old male BALB/c-nu nude mice (Experimental Animal Center of Guangxi Medical University, China) were injected with 1.0 × 10^6^ pCMV6-Entry-5-8F cells in the right flank, and an equal amount of *HMCGL*-5-8F cells was injected into the left flank as a control. The tumor volume was assessed by 2D measurements every 2 days. Tumor volume was calculated as volume (mm^3^) = length × width^2^ × 0.5. Two weeks after inoculation, all mice were killed and tumors were removed. The animal study was approved by the Animal Ethical Committee of First Affiliated Hospital of Guangxi Medical University. All the methods were carried out in accordance with the approved guidelines.

### Wound healing assay

Cells (5 × 10^5^ per well) were seeded into 6-well plates and allowed to adhere overnight with DMEM medium without FBS. Monolayer cells were scratched by using a sterile 200-μl pipette tip. The width of scratch was measured at 3 time points (0, 12 and 24 h) by light microscopy (CKX41, Olympus, Japan). The experiment was performed in triplicate.

### Transwell assay

Cells (2.5 × 10^4^ per well) resuspended in serum-free DMEM medium were seeded in the upper chamber of 24-well Bio-Coat Invasion Chambers (BD, Bedford, MA, USA) coated with Matrigel. The lower chamber was filled with DMEM medium with 10% FBS. Non-invading cells were removed by using a cotton-tipped swab at 48 h. Migratory and invasive cells on the lower membrane surface were fixed with 1% paraformaldehyde, stained with 0.5% crystal violet and photographed.

### Western blot analysis

Proteins were separated by SDS-PAGE gel and transferred to nitrocellulose membranes (HATF00010, Millipore, Ireland), which were blocked with skim milk for 2 h at room temperature, then incubated with primary antibodies overnight at 4 °C followed by secondary antibody for 1 h at room temperature. The blotted proteins were detected and quantified by using a CCD camera in a ChemiDoc XRS instrument (Bio-Rad, USA) with Image Lab software.

### Statistical analysis

All data were analyzed by using SPSS v16.0 (SPSS Inc., Chicago, IL, USA). Data are expressed as mean ± SD and were analyzed by Pearson’s chi-square test and Fisher’s exact test. Statistical significance was considered at *p* < 0.05.

### Data availability

The microarray datasets analyzed during the current study are available in the PubMed repository: https://www.ncbi.nlm.nih.gov/sites/GDSbrowser?acc=GDS3341.

## Electronic supplementary material

supplementary information
