# Supplementary material for: Inactivation of HMGCL promotes proliferation and metastasis of nasopharyngeal carcinoma by suppressing oxidative stress
Source: Sci Rep. 2017 Sep 20;7:11954. doi: 10.1038/s41598-017-11025-2 (PMC5607293; doi:10.1038/s41598-017-11025-2)
Supplement: Supplementary file 1 — supplementary information [file 41598_2017_11025_MOESM1_ESM.pdf]

# Inactivation of HMGCL promotes proliferation and metastasis of nasopharyngeal carcinoma by suppressing oxidative stress

Wenqi Luo[#1], Liting Qin[#1], Bo Li[2], Zhipeng Liao[3], Jiezhen Liang[3], Xiling Xiao[3], Xue Xiao[3], Yingxi Mo[4], Guangwu Huang[3], Zhe Zhang[3], Ping Li[1]* and Xiaoying Zhou*[5]

[1]Department of Pathology, First Affiliated Hospital of Guangxi Medical University, Nanning, China

[2]Department of Radiotherapy, First Affiliated Hospital of Guangxi Medical University, Nanning, China

[3]Department of Otolaryngology-Head & Neck Surgery, First Affiliated Hospital of Guangxi Medical University, Nanning, China

[4]Department of Research, Affiliated Tumor Hospital of Guangxi Medical University, Nanning, China

[5]Life Science Institute, Guangxi Medical University, Nanning, China

#The first two authors contributed equally to this work.

*Correspondence to: Ping Li & Xiaoying Zhou

Supplementary Figure 1

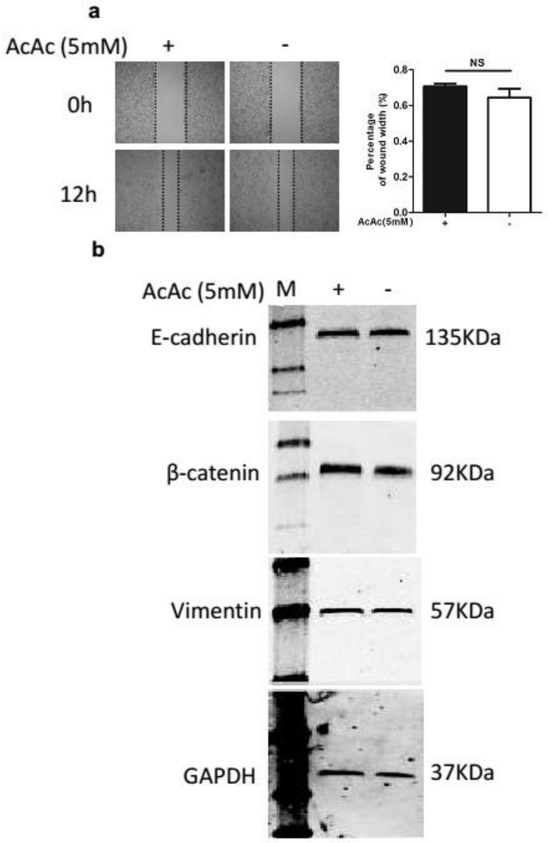


Supplementary Figure Legend 1:

**The motility of 5-8F cells does not affected by acetoacetate (AcAc).** (a) The migration of 5-8F cells upon the treatment of 5mM lithium acetoacetate (Li-AcAc, Sigma Aldrich, # A8509) was analyzed by wound healing assay. (b) Western blot analysis of the expression of three key molecules involved in EMT, E-cadherin, β-catenin and Vimentin. GAPDH was an internal control. Data are mean ±SD (n=3).

Supplementary Figure 2

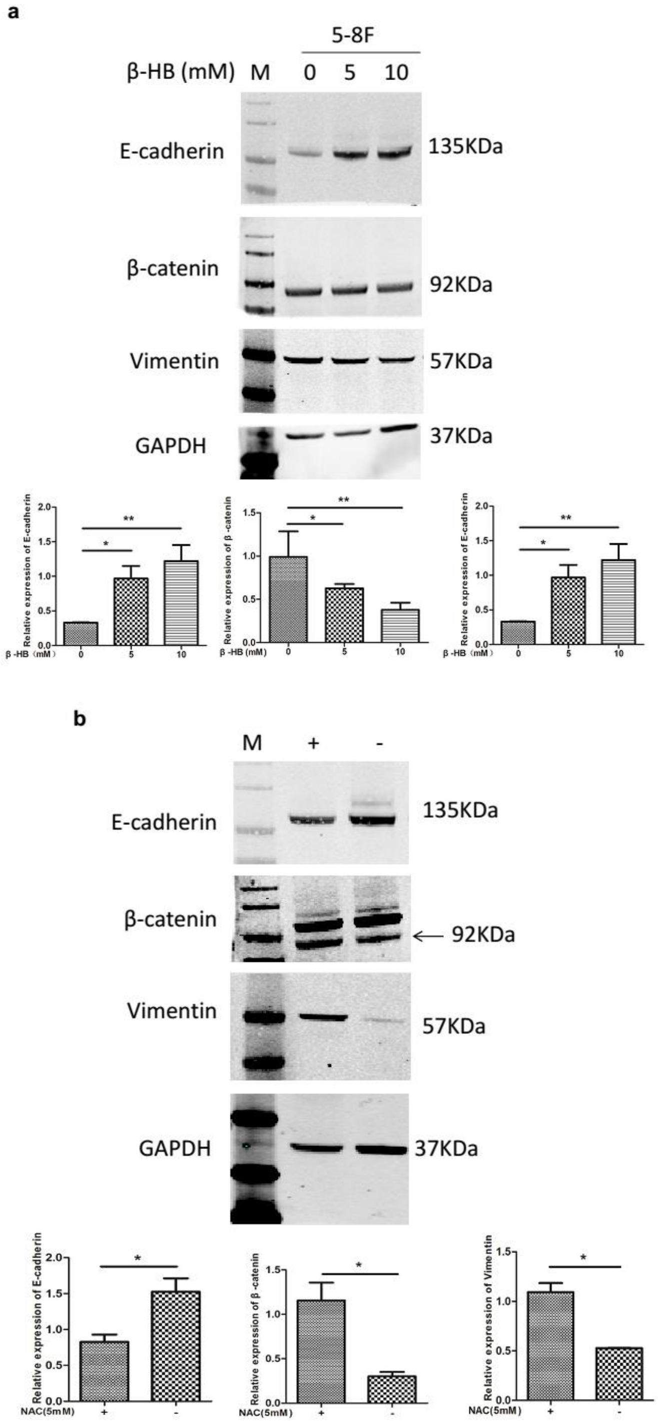


Supplementary Figure Legend 2:

**Exogenous β-HB treatment promotes the mesenchymal-epithelial transition (MET) in 5-8F cells. NAC reversed the MET functioned by HMGCL in HMGCL-5-8F cells.** (a)Western blot analysis of the expression of three key molecules involved in E-cadherin, β-catenin and Vimentin in 5-8F treated by β-HB (0mM, 5mM and 10mM) for

24 h. (b) EMT markers were analyzed by western blot in HMGCL-5-8F cells treated with NAC (0mM, 5mM) for 24 h. GAPDH was an internal control. Data are mean±SD (n=3). (*$p<0.05$; **$p<0.01$)
